# Ensemble and Gossip Learning-Based Framework for Intrusion Detection System in Vehicle-to-Everything Communication Environment

**DOI:** 10.3390/s24206528

**Published:** 2024-10-10

**Authors:** Muhammad Nadeem Ali, Muhammad Imran, Ihsan Ullah, Ghulam Musa Raza, Hye-Young Kim, Byung-Seo Kim

**Affiliations:** 1 Department of Software & Communications Engineering, Hongik University, Sejong-si 30016, Republic of Korea; nadeem@mail.hongik.ac.kr (M.N.A.); royimranpk@gmail.com (M.I.); danish1852@gmail.com (I.U.); ghulammusaraza96@gmail.com (G.M.R.); 2 School of Games/Game Software, Hongik University, Building B, Room # 211, 2639 Sejong-ro, Sejong-si 30016, Republic of Korea; hykim@hongik.ac.kr

**Keywords:** ensemble learning, gossip learning, NIDS, machine learning, data privacy, V2X

## Abstract

Autonomous vehicles are revolutionizing the future of intelligent transportation systems by integrating smart and intelligent onboard units (OBUs) that minimize human intervention. These vehicles can communicate with their environment and one another, sharing critical information such as emergency alerts or media content. However, this communication infrastructure is susceptible to cyber-attacks, necessitating robust mechanisms for detection and defense. Among these, the most critical threat is the denial-of-service (DoS) attack, which can target any entity within the system that communicates with autonomous vehicles, including roadside units (RSUs), or other autonomous vehicles. Such attacks can lead to devastating consequences, including the disruption or complete cessation of service provision by the infrastructure or the autonomous vehicle itself. In this paper, we propose a system capable of detecting DoS attacks in autonomous vehicles across two scenarios: an infrastructure-based scenario and an infrastructureless scenario, corresponding to vehicle-to-everything communication (V2X) Mode 3 and Mode 4, respectively. For Mode 3, we propose an ensemble learning (EL) approach, while for the Mode 4 environment, we introduce a gossip learning (GL)-based approach. The gossip and ensemble learning approaches demonstrate remarkable achievements in detecting DoS attacks on the UNSW-NB15 dataset, with efficiencies of 98.82% and 99.16%, respectively. Moreover, these methods exhibit superior performance compared to existing schemes.

## 1. Introduction

The dream of autonomous vehicles (AVs) is now a reality that will become an ordinary commodity in everyone’s life soon. AVs have introduced a new era of usage, automation, planning, and management for autonomous vehicle systems (AVS) [1], also setting new dimensions for smart city infrastructure and stringent quality of service (QoS) requirements by communication systems to form intelligent transport systems (ITSs) [2,3]. Communication systems like 4G, 5G, 5G Beyond, 6G, and Information Communication Networks (ICNs) [4] have undergone remarkable evaluation to enable vehicle-to-everything (V2X) communication to ensure end user comfort and, most importantly, safety [5]. However, due to the tremendous growth of AVs, the vulnerabilities of the safety and security of autonomous vehicles have also risen to a new level and have gained much attention from design engineers of smart vehicles and communication systems [6]. The incapability of the traditional security solution for autonomous vehicles, especially for vehicle-to-vehicle (V2V), vehicle-to-infrastructure (V2I), vehicle-to-pedestrian (V2P), and vehicle-to-everything (V2X) communication, requires finding the most appropriate and robust security solutions to detect and mitigate any security threat to the aforementioned systems [7]. Various reports have shown the efforts of cybercriminals to launch remote attacks to overwhelm V2X systems in order to cause the braking and engine systems to malfunction [8]. Cybercriminals can cause property damage to vehicles or even physical harm to passengers by exploiting vulnerabilities in autonomous vehicle (AV) systems or by performing man-in-the-middle attacks on communication networks. To prevent such incidents, a foolproof security system for AVs is essential to avoid malfunctioning and mitigate any potential cyber-attacks targeting communication systems. Researchers, engineers, and the business community have made remarkable advancements in AV technology, focusing on efficient connectivity and a smooth driving experience.

Given the risk of cybercrimes, the primary concern is ensuring human safety in the event of AV malfunction or service denial by the roadside unit (RSU) that serves as the AV’s server [9]. Service provision is a critical aspect of AVs, as it offers a comprehensive range of services required throughout the vehicle’s journey, including enhanced mobile broadband (eMBB) and ultra-reliable low-latency communication (URLLC) for both entertainment and mission-critical scenarios, as introduced in 5G, 5GB, and 6G technologies [10]. The unavailability of these services can pose serious threats to the safety of AV passengers and result in vehicle damage. A failure of the RSU to provide these services is referred to as a denial of service (DoS), one of the most well-known cyber-attacks on AVs. Addressing this issue necessitates the development of security systems capable of detecting and mitigating such attacks.

Recently, artificial intelligence (AI), machine learning (ML), deep learning (DL), and transfer learning have offered powerful frameworks for detecting cybercrimes through regression- and classification-based methods [11,12]. These methods include algorithms such as Naive Bayes, convolutional neural networks (CNNs), decision trees (DTs), and support vector machines (SVMs), which provide robust classifications distinguishing normal traffic flow from cyber-attack traffic. Furthermore, these architectures also comprise various layers, such as data preprocessing, features, and model selection [13]. These detection and mitigation systems are commonly referred to as intrusion detection systems (IDSs) [14].

### Background and Motivation

**Figure 1:**
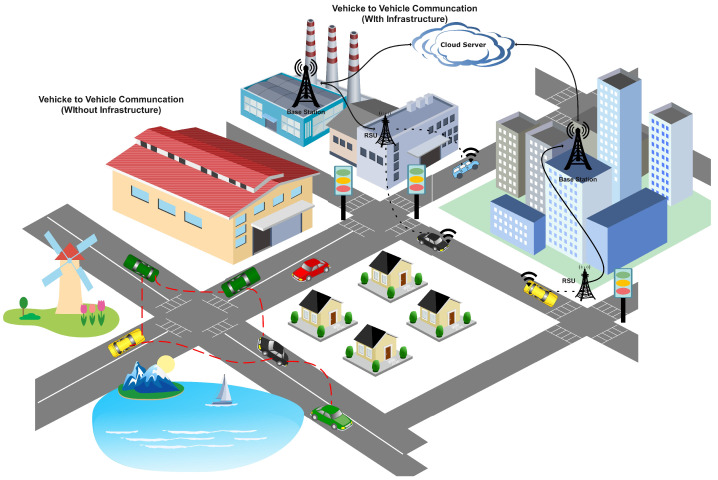
Autonomous vehicle environment with/without infrastructure.

AVs work via two modes of communication provided by the 3rd Generation Partner Project (3GPP), the standard body of the vehicle to everything (V2X), i.e., Mode 3 and Mode 4, as illustrated in Figure 1 above. In Mode 3, the AVs communicate with existing infrastructure, like roadside units, for any service provisioning, while in Mode 4, the AVs use the wireless ad-hoc vehicle environment (WAVE) to communicate with each other in the absence of the infrastructure [15,16]. The coexistence of Mode 3 and Mode 4 is the main motivation to provide two solutions that encounter ensemble learning for the Mode 3 situation, in which the RSU ensembles the locally trained model through a voting mechanism after acquiring the locally trained model of each associated AV in its range. For the Mode 4 scenario, we proposed a gossip learning (GL)-based solution, in which the AVs will share locally trained models, and the AV will decide to retain a specific model of future DoS attacks among all the shared models after testing the each received model using its testing data. The core contributions of this study are as follows.

1. Autonomous vehicle systems operating in Mode 3 and Mode 4 are considered for the development of distinct IDS solutions tailored to each mode.
2. An ensemble learning method is proposed for Mode 3, which is based on the voting mechanisms, and selects the prediction outcome based on the majority vote among the received prediction of each locally trained model at RSUs by the AVs.
3. A gossip learning scheme is proposed for Mode 4, in which the AVs build a pool of trained models by sharing their locally trained models and decide to choose the most suitable trained model based on the best accuracy of the trained models.
4. Extensive simulations are performed using the UNSW-NB15 dataset, which contains both real normal traffic and synthesized attack traffic. The performance of the proposed schemes is evaluated based on classification accuracy (CA) and misclassification rate (MCR). Furthermore, various statistical parameters, including sensitivity, specificity, false positive rate, false negative rate, and precision, are also used to assess the effectiveness of the systems.

The rest of the paper is structured as follows: Section 2 provides a comprehensive literature review of existing schemes in developing IDSs for AVs, Section 3 provides a detailed description of the proposed scheme, including SVM- and ANN-based DoS attack detection in an AV environment, Section 4 presents a description of the parameters utilized for the evaluation of the proposed scheme, Section 5 discusses the simulation results, and finally, our conclusions and future work are presented in Section 6.

## 2. Literature Review

DoS attacks work by making extensive requests, overburdening the AV’s processing capabilities and resulting in the AVs exhibiting unresponsive behavior. This unresponsiveness or denial of service results in serious issues, such as accidents, or in the worst case, loss of life [17].

In [18], the author used ML-based gradient boosting methods to detect invalid data flow to defend against AVs cyber-attacks. The core contribution of the study was in recognizing inappropriate pieces of information used in AVs and classifying them into cyber-attacks or normal information. Rajendar and Kaliappan, [19] use a modified CNN (M-CNN) to implement an IDS along with Safety Pilot Model Deployment (SPMD). In addition to this, the IDS detection was aided by a support vector machine (SVM) and an isolation forest (IF) to enhance its detection accuracy. The M-CNN was able to fully capture the possible variation in the AVs’ data with the help of the efficient layer and ReLU activation functions.

In [20], Al-Hawawreh and Hossain implement an IDS by combining distribution-based learning for AVs. The authors trained a local model and exchanged the trained model with a satellite mesh network, and also took care to preserve privacy while model sharing. In [21], the author proposed particle swarm optimization (PSO) and an adaptive genetic algorithm (AGA) to analyze android malware detection for AVs. The author utilized PSO for feature selection and then used XGBoost and random forest (RF) to improve its detection accuracy.

Pawar et al. [22] proposed DT and K-nearest neighbor (KNN) algorithms to detect false communications from devices to secure controlled network (CAN) vehicles. In [23], Berry et al. used ML-based XGBoost techniques to identify inappropriate information used in vehicles that cause cyber-attacks on AVs. In [24], an IDS was implemented to protect AVs from messages from the outside world using a smart device by incorporating the Proportional Overlapping Score (POS) model for DoS attacks. Nazarduddin and Chaudry [25] provide assessment standards to classify robust ML methods of detecting cybercrimes. The authors also provide an insight into AV architecture, cyber security, AV potentials, and expected dangers to AVs in the future.

In [26], the author employed a support vector machine to monitor point-to-point critical path monitoring (P2PCPM) to detect attack nodes and provide seamless communication to AVs. The author demonstrated the proposed scheme’s effectiveness for 1000 nodes, showing its capability for scalability for the future. In [27], the author proposed an IDS scheme utilizing a software-defined network (SDN) (OpenFlow 1.0, OvS 2.7 with the Ubuntu kernal version 4.4.) to detect DoS attacks by continuously scanning the port. The author utilized model-based prediction for the future behavior of AVs to detect and mitigate DoS attacks.

Hybrid model-based machine learning algorithms also play a vital role in detecting and defending against DoS attacks in AVs [28,29,30]. In [28], a hybrid model that combines a deep neural network for feature extraction and an SVM classifier with its variants is utilized for attack classification. Besides machine learning algorithm selection, feature selection is also a critical aspect while developing such IDSs [29]. In [31], the author demonstrates the effectiveness of pre-processing techniques, the selection of features, and the real-time monitoring of IDSs.

K. Huang et al. [32] proposed a federated architecture for the Internet of Vehicles (IoV) that enables collaboration with distributed edge devices to detect attack traffic aimed at IoVs while preserving data traffic privacy. The authors evaluated the proposed scheme using benchmark datasets, including CICIDS2017 and CAN-Intrusion. The results demonstrated a significant detection accuracy of 98.51% for the CAN-Intrusion dataset and 97.74% for the CICIDS2017 dataset. Additionally, the authors measured the prediction latency, which was found to be under 10 milliseconds, making the system ideal for deployment in IoV environments, even on devices with limited computational resources.

In [33], the author emphasizes the importance of data privacy and ownership control when sharing information among devices, thereby maximizing the potential benefits of data-driven applications. Data sharing is a key element in various learning paradigms, such as federated learning and the ensemble learning approach proposed in this paper. The author suggests a robust mechanism that stores data on an external server and incorporates a blockchain framework to maintain a transaction record of the data while sharing the data among devices. This proposed framework not only provides excellent data privacy but also guarantees remarkable ownership control. Furthermore, it accommodates a diverse range of data types, making it highly suitable for various applications.

## 3. Proposed Scheme

**Figure 2:**
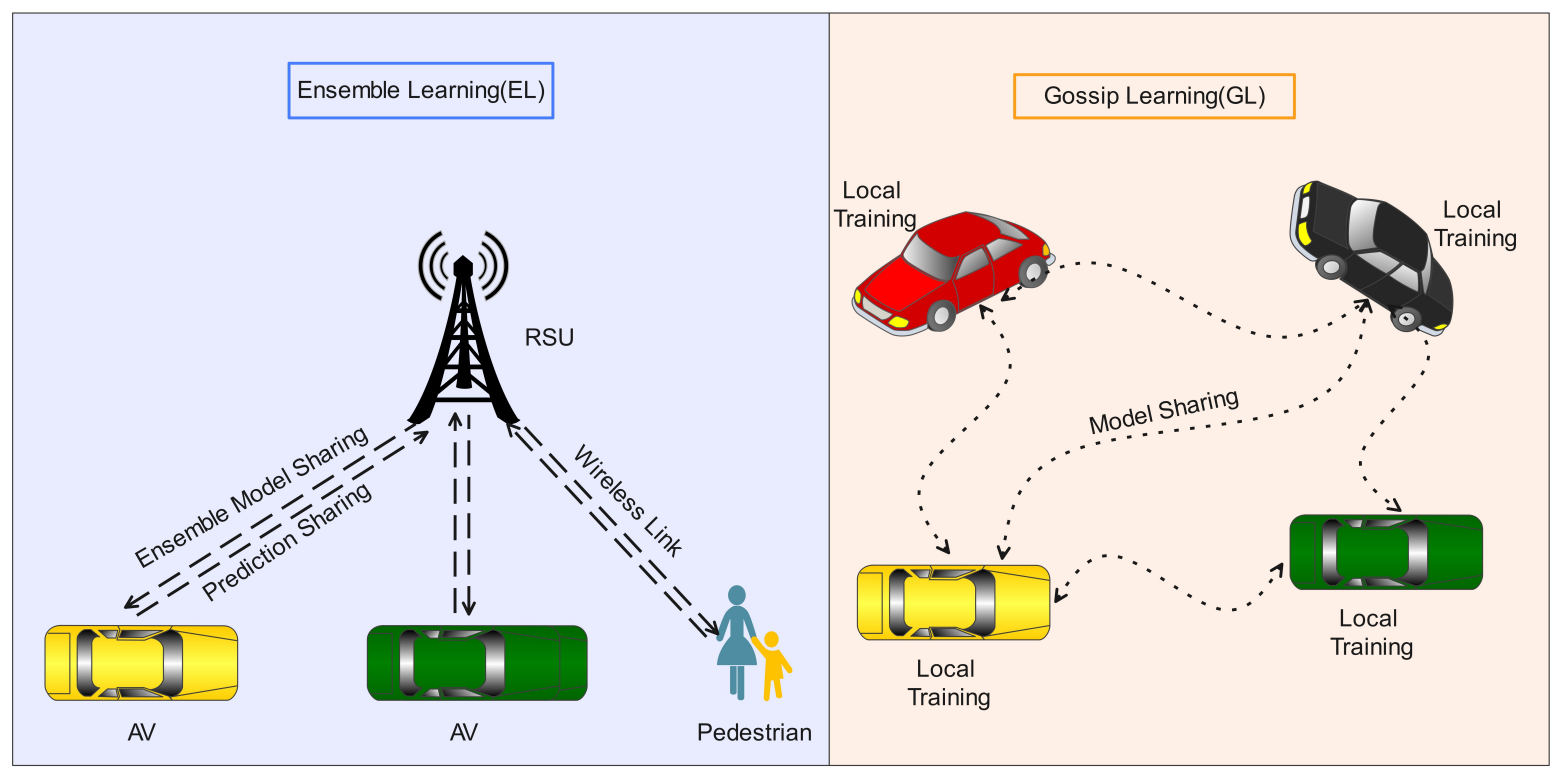
EL and GL-based proposed solutions for Mode 3 and Mode 4 in V2X environment.

In this section, we will provide a brief description of the proposed architecture for DoS attack detection using ensemble learning (EL) and gossip learning (GL) frameworks for the Mode 3 and Mode 4 V2X. To fully understand the situation, let us consider an AV environment as shown in Figure 2 below. A vehicle $V_{i}$ is traveling in the city, and during its journey, it remains connected with a *j*th RSU. The AV always remains connected to the RSU to access certain services, known as Mode 3 in V2X, which is backed up by the infrastructure. For Mode 3, we propose ensemble learning, which acquires the predictions of each AV connected with it and decides on an optimal detection result using a voting scheme, as shown in Figure 1.

The main concept of ensemble learning is to combine weak learner models (trained locally) to enhance the prediction performance of each model by deciding the prediction outcome based on the majority. This is directly related to the possible bias of any trained model during its prediction performance. The RSU in Mode 3 ensembles each AV’s local model’s prediction after receiving them. Each AV in the environment is required to send only its prediction results rather than sharing the weights of the trained model or the local data, as the existing scheme requires federated learning (FL) [34] or data fusion schemes [35]. For ensemble learning, we utilized support vector machine (SVM) and artificial neural network (ANN) algorithms to train the local models and share their predictions with the RSU for ensemble purposes. The rationale for selecting multiple variants of the SVM classifier [36] and the ANN algorithm [37] lies in their respective strengths. SVM classifiers demonstrate significant performance with high-dimensional datasets, while the ANN algorithm excels in handling complex, non-linear, and large datasets. By incorporating both the SVM classifier and the ANN algorithm, the scalability of our proposed system is enhanced, enabling it to manage datasets of varying sizes and complexities effectively. AVs may experience a situation that lacks the traditional infrastructure, for example, if a vehicle is visiting a lake, the coast, the desert, or mountains; then, vehicles are required to communicate with each other rather than a central infrastructure such as an RSU. In that case, AVs follow the Mode 4 operation of V2X. For Mode 4 operation, we propose a gossip learning (GL)-based solution, in which AVs are required to exchange locally trained models with each other. AVs build a pool of locally trained models and test each model using their testing data. After testing their data, the AVs sort each received model’s accuracy in descending order and select the specific model with the highest accuracy, as depicted in Figure 2 above. For GL, models locally trained by SVM and ANN are shared among the AVs.

**Figure 3:**
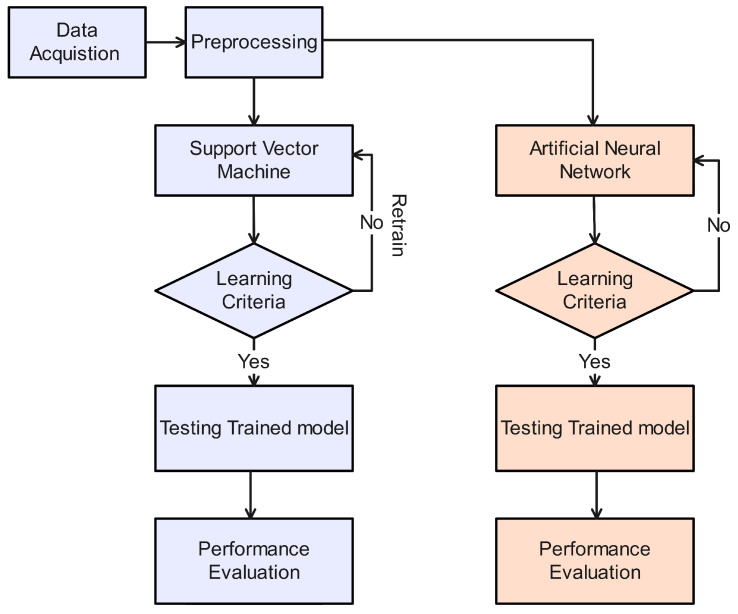
SVM- and ANN-based DoS attacks in AVs.

Figure 3 above illustrates the mechanism of DoS attacks in AVs using the support vector machine (SVM) and artificial neural network (ANN) algorithms. To implement both schemes, the first step is to acquire data related to normal traffic and attack traffic in the AV scenarios. After acquiring the data, the preprocessing phase is applied to the collected data. In the preprocessing phase, we check for missing values, perform normalization, and remove duplicate values. After performing the preprocessing step, the refined data are fed into the SVM classifier and ANN algorithm. Both the SVM classifier and the ANN algorithm are utilized for AV traffic classification.

A stop criterion is set during the training of the SVM classifier, which is based on the number of iterations or the mean square error (MSE) rate. After meeting the stop criteria, the trained model of both classifier models is obtained and then subjected to data testing for further prediction. After a successful prediction, the prediction performance of both testing models is further evaluated using performance parameters such as classification accuracy (CA), misclassification rate (MCR), sensitivity, specificity, negative partial value (NPV), false positive rate (FPR), false negative rate (FNR), and precision.

### 3.1. SVM-Based DoS Attack Classification

**Figure 4:**
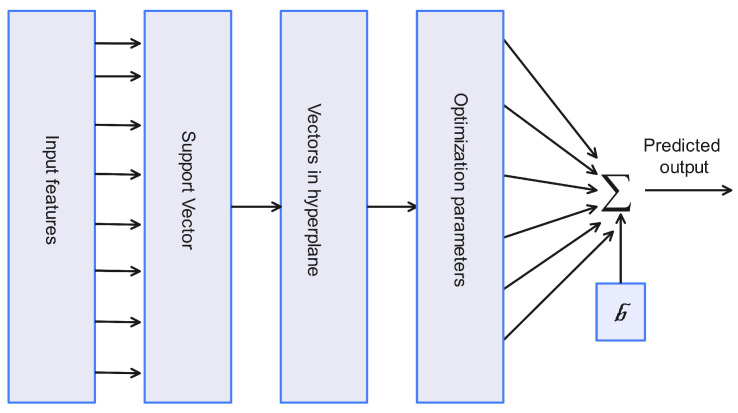
SVM-based DoS attack in AVs.

Figure 4 illustrates the SVM classifier. In the SVM classifier, the input features are classified using the hyperplane, which is separated using a slope line. The slope line for higher-dimensional data can be expressed using Equation (1) given below. In Equation (1), *y* and *x* are two points, *m* is the slope, and *c* is the intercept.

(1) $$y-y_{1}=m\left(x-x_{1}\right)+c$$

The resultant vector *r* can be computed using the dot product of vectors $\vec{y}$ and $\vec{x}$. This involves multiplying corresponding components, $y_{i}$ and $x_{i}$, for each point *i*, and summing the resulting overall *k* points, as expressed in Equation (2).

(2) $$r=\vec{y}.\vec{x}=\sum_{i=1}^{k}y_{i}x_{i}$$

This *r* acts as the Liner kernel function and can be expressed as Equation (3), and the hyperplane is defined in Equation (4) to make a prediction. In Equation (4), if the points are above 0, then the traffic is classified as class 1 (attack traffic); otherwise, it is classified as class 0 (normal traffic).

(3) $$K\left(y_{i},y_{j}\right)=y_{i}.y_{j}$$

(4) $$H\left(r_{i}\right)=\left\{\begin{array}{cc} 1 & \begin{array}{cc} if & r.y+c\geq 0 \end{array}\\ 0 & otherwise \end{array}\right.$$

### 3.2. ANN-Based DoS Detection

**Figure 5:**
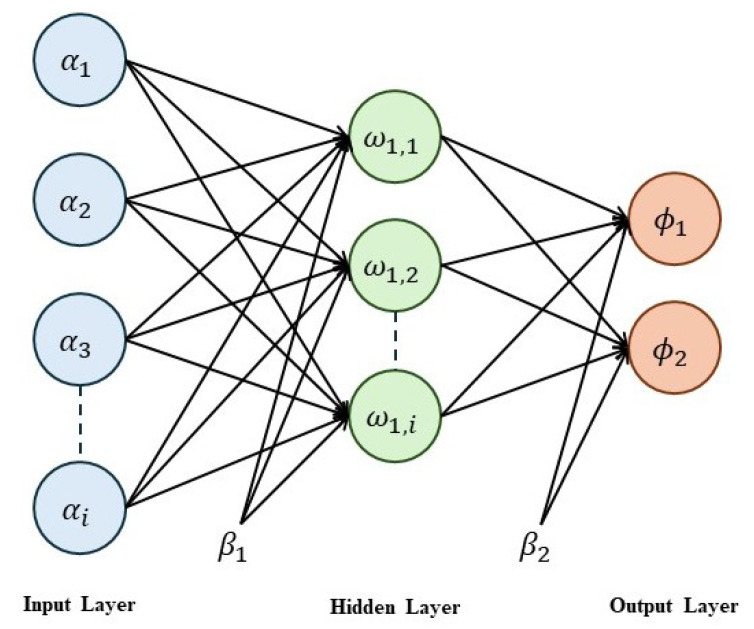
ANN’s architecture for DoS attack detection in AVs.

We employ an artificial neural network (ANN) for AV local training, comprising input, hidden, and output layers, as shown in Figure 5. The ANN architecture consists of 25 layers with validation and testing data of 15% each. The input layer comprises multiple neurons, which are connected to each neuron of the hidden layer, and similarly, the hidden layer neurons are linked to the output layer. At each layer, the output can be calculated after passing the neuron’s current value through an activation function. In the ANN, we utilized the scaled conjugate gradient (SCG) as a training algorithm. We also trained the ANN model using another training algorithm, such as Levenberg–Marquardt (LM) or Bayesian Regularization (BR); however, the most suitable results were achieved by the SCG training algorithms. The output of the $i_{th}$ hidden layer’s neuron can be calculated using Equation (5) below. In Equation (5), $\omega 1_{j,i}$ represents the weight of the *i*th neuron of *j*th hidden layer, $\alpha_{i}$ represents the *i*th input features, and $\beta_{1}$ and $\beta_{2}$ represent the biases of the hidden and output layers, respectively.

(5) $$HL_{j}^{V_{i}}=\frac{1}{1+e^{-(\sum_{i=1}^{m}\omega 1_{j,i}^{V_{i}}\alpha_{i}+\beta_{1})}}$$

In Equation (6), $\phi_{j}^{V_{i}}$ represents the output of the jth neuron of the output layer for vehicle $V_{i}$, and $\beta_{2}$ represents the bias of the output layer. After obtaining the output $\phi_{j}^{V_{i}}$ from the output layer, the predicted output is compared with the targeted output $TO_{k}^{V_{i}}$, as given in Equation (7), to calculate the error $E_{V_{i}}$ between the predicted and target outputs.

(6) $$\phi_{j}^{V_{i}}=\frac{1}{1+e^{-(\sum_{i=1}^{m}HL_{j}^{V_{i}}\omega_{i}+\beta_{2})}}$$

(7) $$E_{V_{i}}=\frac{1}{2}{\sum_{k=1}^{q}\left(TO_{k}^{V_{i}}-\phi_{k}^{V_{i}}\right)}^{2}$$

If the learning criteria are not met, then the weights are back-propagated to the hidden layer. The weights of the hidden layers are updated using the current weights $\omega 1_{j,k}^{V_{i}}\left(t\right)$ by adding the change in weights, which is measured using the $\Delta \omega 1_{j,k}^{V_{i}}$ multiplied by some constant factor η. The updated weights are represented by $\omega 1_{j,k}^{V_{i}}\left(t+1\right)$ for the hidden layer using Equation (8).

(8) $$\omega 1_{j,k}^{V_{i}}\left(t+1\right)=\omega 1_{j,k}^{V_{i}}\left(t\right)+\eta \Delta \omega 1_{j,k}^{V_{i}}$$

Upon updating the weights of the hidden layer, all weights are collected in a matrix form known as the trained model of the ANN algorithm and further utilized for the testing phase.

### 3.3. Voting Ensemble Learning (VEL)

**Figure 6:**
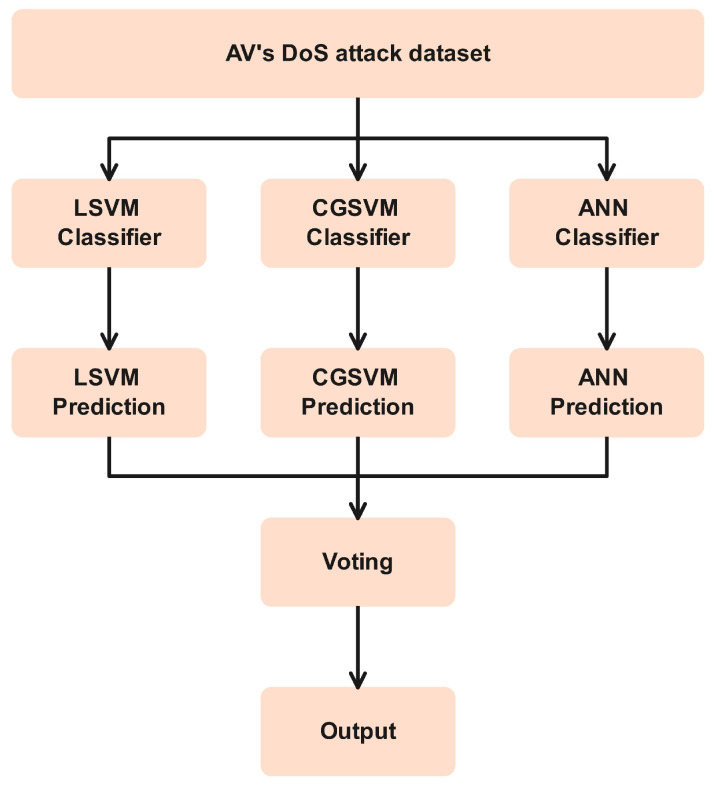
Voting-based ensemble learning architecture.

Ensemble learning offers a unique mechanism that is based on a voting mechanism to combine the power of various classifiers for classification purposes, as shown in Figure 6. In voting-based ensemble learning (VEL), diverse classifier models are incorporated for the classification of a subjected dataset, and we utilized the autonomous vehicles dataset for the DoS attack [38]. The classifier trains the model based on the instances and the features of the dataset; then, predictions are performed using an unseen testing dataset. Upon obtaining the diverse prediction performance, all the predictions are concatenated into a matrix to implement the voting mechanisms on the combined predictions. The voting mechanisms then observe each prediction of these diverse classifier models and finally select the best prediction based on the majority among them [39]. Additionally, VEL also enhances the robustness of the prediction performance by eliminating the possible bias and variance in individual classifier prediction performance [40].

### 3.4. Gossip Learning (GL)

**Figure 7:**
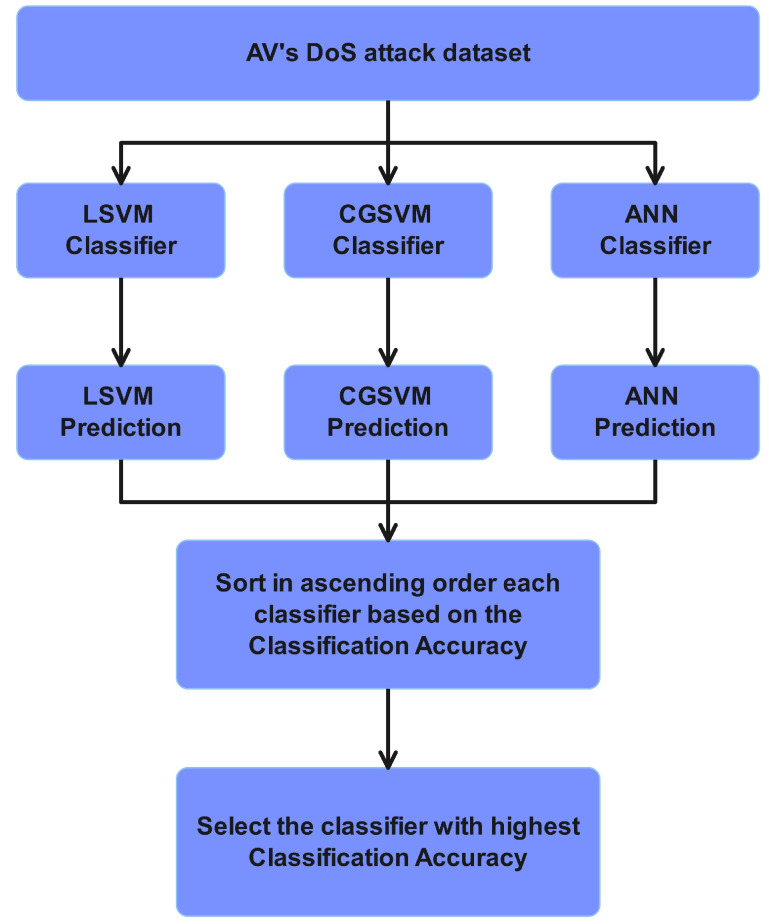
Gossip learning (GL)-based classifier model sharing.

Gossip Learning (GL) is a decentralized framework in which each autonomous vehicle (AV) shares its locally trained model with other AVs, allowing them to collectively build a diverse pool of models. This process eliminates the need for a central controller, resulting in a fully distributed autonomous system. A flowchart of the GL mechanism is depicted in Figure 7, where AVs exchange their locally trained models using Mode 4 communication. Each AV then evaluates the received models using its dataset to test the performance of the classifiers. After testing each received model, the AVs sort each classifier model in an ascending order to identify weak models. After sorting, the AVs select a classifier model for future prediction. GL offers a sharing mechanism to for AVs to exchange locally trained models among themselves. The reason for such sharing is to resolve AVs’ issues, such as low computing power, bias and variance in the training model, and an inability to collect diverse datasets.

## 4. Evaluation Parameters

This section provides comprehensive definitions of the parameters used for the evaluation of the aforementioned schemes.

Classification accuracy (CA) measures the percentage of overall correctly detected class instances, as shown in Equation (9) [41]. Higher CA values show better capability of the proposed system in a real-time environment.

(9) $$CA=\frac{TP+TN}{TP+FN+FP+TN}*100$$

The Misclassification rate (MCR) provides the percentage of wrongly predicted instances. A lower MCR value shows better robustness of the trained model against unseen data samples. The MCR can be computed using Equation (10).

(10) $$MCR=100-\frac{TP+TN}{TP+FN+FP+TN}*100$$

Sensitivity is also known as the true positive rate (TPR) and can be measured using Equation (11). Sensitivity is used to measure the efficiency of the trained model to predict true instances only.

(11) $$Sensitivity=\frac{TP}{TP+FN}*100$$

Specificity measures the proportion of correctly negative instances and is also known as the true negative rate (TNR), as presented in Equation (12). This value should be higher for a well-trained model.

(12) $$Specificity=\frac{TN}{TN+FP}*100$$

The NPV measures correctly predict negative instances as negative. The NPV can be computed using Equation (13), which comprises the ratio of true negatives (TNs) to the sum of true negatives (TNs) and false negatives (FNs).

(13) $$NPV=\frac{TN}{TN+FN}*100$$

FPR provides a measure of false positive prediction by the trained model and can be computed using Equation (14). Lower values of FPR show higher classification accuracy.

(14) FPR=100−NPV

FNR provides a measure of false negative prediction and can be computed using Equation (15), and should also have a smaller value for a well-trained model.

(15) FNR=100−Sensitivity

Precision is computed to provide the ratio of true instances over true positive and true negative instances using Equation (16). This represents the ability of the trained model to correctly predict a true instance as a true instance. A higher value of precision demonstrates higher performance efficiency of the trained model.

(16) $$Precision=\frac{TP}{TP+FP}*100$$

## 5. Simulation Results

### 5.1. Simulation Environment

For the simulation environment, we utilized MATLAB 24a running on a Windows 11 platform with 16GB of RAM and a Core i7-11700 processor. In our simulations, we simulated the ANN algorithm and SVM classifier (linear SVM, coarse Gaussian SVM) and utilized multiple-trained models for the ensemble learning and gossip learning framework. To fully observe the effectiveness of ensemble learning, we simulated two SVM variants and ANN algorithms, which were utilized in the voting mechanism of ensemble learning.

### 5.2. UNSW-NB15 Dataset

We utilized the UNSW-NB15 dataset, developed by the Cyber Range Lab at the Australian Centre for Cyber Security (ACCS), University of New South Wales (UNSW) [42]. This dataset consists of nominal, numeric, and timestamp values for various features. The raw network traffic was captured using the IXIA Perfect Storm tool, which contains both normal and attack traffic packets. The features were generated using the Argus and Bro-IDS tools, along with various algorithms, including the class labels. Each sample was labeled as either normal traffic (class 0) or attack traffic (class 1), providing a binary classification of the output features. In our simulation, the UNSW-NB15 dataset included 100,574 instances with 43 features. For training, we used 76,500 instances (70% of the dataset), leaving 24,074 instances for testing. The training set consisted of 42 input parameters, with the 43rd feature serving as the output parameter.

### 5.3. Training Performance

**Figure 8:**
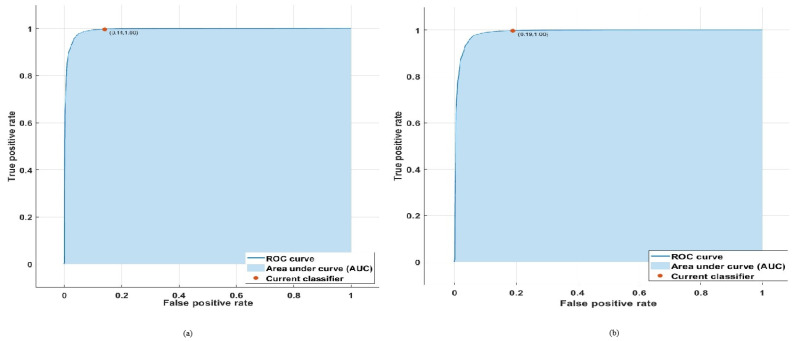
(**a**) linear SVM training ROC curve. (**b**) coarse Gaussian SVM training ROC curve.

In this section, we will provide comprehensive details on the training performance of the LSVM, CGSVM, and ANN algorithms. The training performance of the LSVM and CGSVM classifiers were determined using a receiver operating characteristic (ROC) curve, while the ANN algorithm performance was assessed using a mean square error (MSE) graph. Figure 8a,b show the training performance of the linear SVM (LSVM) and coarse Gaussian SVM (CGSVM) in the form of an ROC. The LSVM and CGSVM show higher training performance as both contain a large portion of the dataset under the curve area, showing remarkable training performance. The training ROC curve shows the capability of the LSVM and CGSVM models to classify the normal and attack traffic, with accuracy of 99.9% and 98.8%, respectively. In Figure 8a, the LSVM classifier demonstrates a false positive rate (FPR) of 0.14 (14%), as indicated on the x-axis, and a true positive rate (TPR) of 1.00 (100%), as shown on the y-axis. This high TPR highlights the exceptional ability of the LSVM classifier to accurately identify true positives while maintaining a relatively low FPR. Similarly, in Figure 8b, the CGSVM classifier achieves an FPR of 0.19 (19%) and a TPR of 1.00 (100%). While both classifiers exhibit perfect true positive rates, the CGSVM shows a slightly higher FPR compared to the LSVM.

**Figure 9:**
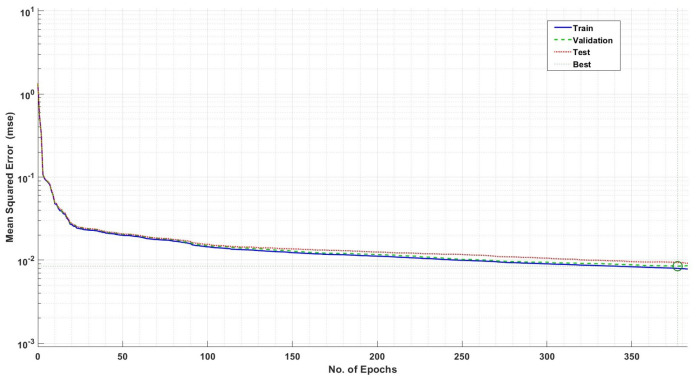
ANN-based model training for DoS attack in AVs.

Figure 9 shows the ANN-based model’s training performance for the AV DoS attack dataset [42]. The mean square error (MSE) graph shows robust performance by continuously minimizing the error between the predicted and actual outputs. We also take care of model overfitting and underfitting during training of the ANN algorithm by splitting the subjected dataset into training (70%), validation (15%), and testing (15%), as shown in Figure 9. In Figure 9, the solid blue line shows the training, the dashed green line shows the validation and the dotted red line shows the testing curve respectively. The training algorithm used in the training of the ANN scheme is the scaled conjugate gradient (SCG) and achieves a training accuracy of 91.79%, validation accuracy of 91.02%, and testing accuracy of 90.54%. It is also important to note that, this testing and validation is solely used for model underfitting and overfitting during the training of the ANN algorithm; however, for the actual testing of the dataset, we utilize the holdout testing mechanism.

**Figure 10:**
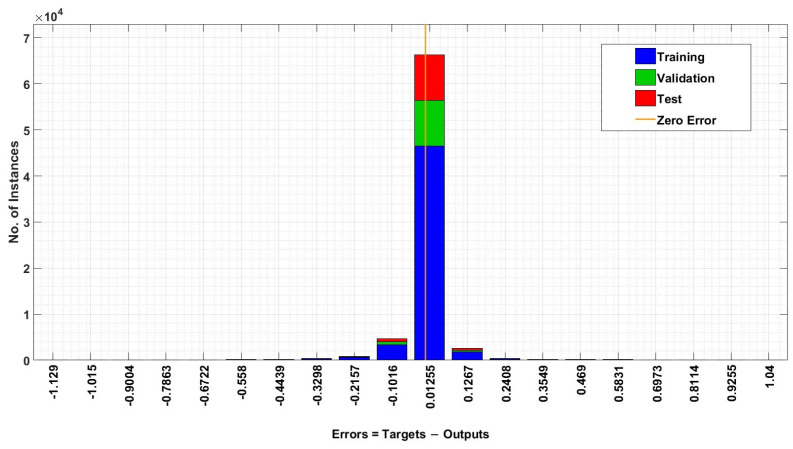
Error Histogram graph of ANN algorithm.

Figure 10 illustrates an error histogram graph of the ANN training model, which shows the occurrence of instances around the zero error line, which is shown as a yellow colored line. Figure 10 depicts the high concentration of the training, validation, and testing instances at the zero error line. The error histogram graph also reveals the skewed behavior of instances around the zero line, which was determined through another analysis of the performance of the training model.

### 5.4. Testing Performance

Testing is an important part of evaluating the trained model, in which unseen data are provided to the trained model to measure the true prediction performance of the trained model.

**Figure 11:**
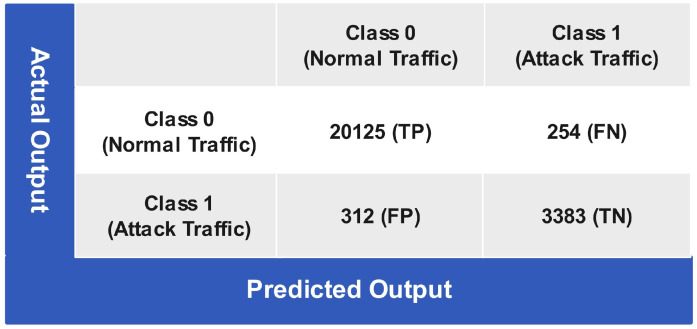
Confusion matrix of linear SVM testing.

Figure 11 shows the testing of the LSVM-trained model, in which the 20125 instances are correctly predicted as class 0 (normal traffic), and they belong to class 0 (normal traffic), referred to as a true positive (TP). However, 254 instances are wrongly predicted as class 1 (attack traffic), while they belong to class 0 (normal traffic), referred to as a false negative (FN). The LSVM predicts 312 instances as class 1 (attack traffic), while they belong to class 0 (normal traffic) known as a false positive(FP), and correctly predicts 3383 instances as class 1 (attack traffic) members, known as a true negative(TN). The LSVM attains an overall classification accuracy (CA) of 97.64%, misclassification rate (MCR) of 2.35%, sensitivity of 98.75%, specificity of 91.55%, negative partial value (NPV) of 93.01%, false positive rate (FPR) of 10.91%, false negative rate (FNR) of 3.27%, and precision of 96.51%.

**Figure 12:**
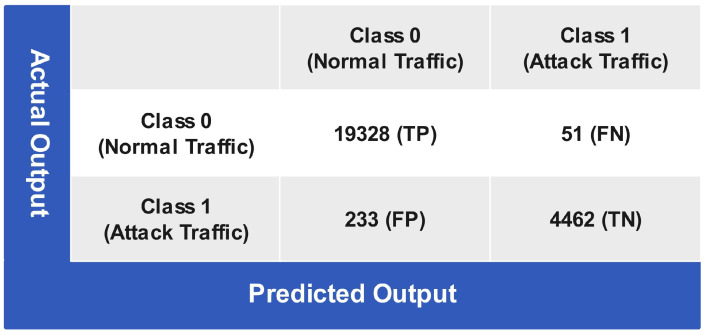
Confusion matrix of coarse Gaussian SVM testing.

Figure 12, shows the testing performance of the coarse Gaussian SVM (CGSVM) algorithm in the form of a confusion matrix. The confusion matrix shows that 19,328 instances are accurately predicted, while 51 instances are predicted as class 1 (attack traffic) instances while belonging to class 0 (normal traffic). For class 1 (attack traffic), 233 instances are wrongly predicted that actually belong to class 0 (normal traffic), and 4462 instances are correctly classified. The statistical performance of the CGSVM classifier is given in Table 1. The CGSVM classifier attains an overall classification accuracy (CA) of 98.82%, misclassification rate (MCR) of 1.17%, sensitivity of 99.73%, specificity of 95.03%, NPV of 98.86%, FPR of 4.96%, FNR of 0.26%, and precision of 98.80%.

**Figure 13:**
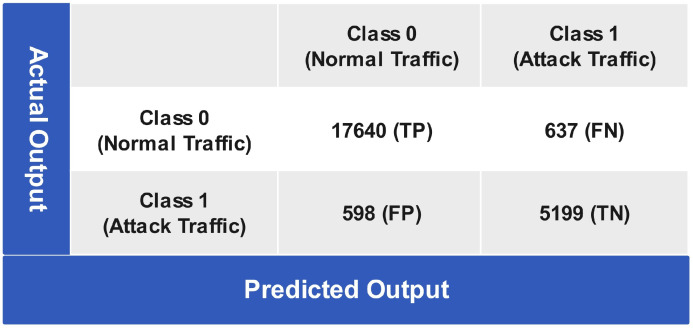
Confusion matrix of ANN algorithm testing.

Figure 13 below shows the prediction performance of the ANN algorithm, in which 17,640 instances are true positive predictions while 637 instances are false negative predictions of class 0 (normal traffic). For class 1 (attack traffic), 598 are false positive predictions, since, in reality, they belong to class 0 (normal traffic), and 5199 instances are true negative predictions, meaning they are correctly predicted. The statistical evaluation of the ANN algorithm provides the performance measurements given in Table 1. The ANN algorithm achieves a CA of 94.86%, MCR of 5.13%, sensitivity of 96.72%, specificity of 89.08%, NPV of 89.68%, FPR of 10.91%, FNR of 3.27%, and precision of 96.51%.

**Figure 14:**
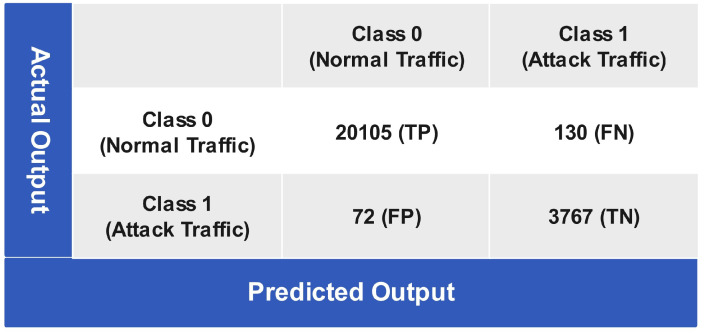
Confusion matrix of VEL testing.

Figure 14, illustrates the prediction performance determined through a voting mechanism by combing the aforementioned classifiers, i.e., the ANN algorithm, LSVM, and CGSVM classifiers. In the VEL mechanism, the decision is made based on the majority of the classifier prediction values. After incorporating the voting mechanism, 20,105 instances are correctly classified as true positives, while 130 instances are misclassified as false negatives for class 0 (normal traffic). For class 1 (attack traffic), only 72 instances are wrongly predicted as false positives, while 3767 instances are correctly predicted as true negatives. The reason for such high performance of the VEL mechanism is its capability to eliminate possible bias and variance in the prediction outcomes of various classifiers.

### 5.5. Result Discussion

The statistical performance of the ANN, LSVM, CGSVM, and VEL algorithms is presented in Table 1, which also offers insights into the attack detection capabilities of the trained models. Among these, the LSVM algorithm demonstrates the highest sensitivity, indicating its robustness in detecting potential DoS attacks. On the other hand, the VEL algorithm achieves the highest specificity, demonstrating its strong ability to minimize false positive detections. The CGSVM classifier stands out with the highest NPV of 98.86%, showcasing its effectiveness in correctly identifying normal traffic. Additionally, the VEL model reports the lowest false positive rate (FPR) of 1.87%, reflecting its capacity to reduce the incorrect classification of normal traffic as attacks. Moreover, VEL achieves the lowest false negative rate (FNR), highlighting its proficiency in detecting attack traffic without leaving a significant portion undetected. Furthermore, the VEL model attains the highest precision value of 99.64%, confirming its ability to resist being overwhelmed by false alarms.

The VEL scheme enhances prediction performance by combining the prediction ability of each classifier model. VEL’s prediction performance is compared with the existing techniques in the literature in Table 2. VEL exhibits a higher classification accuracy of 99.16%, with a minimal misclassification rate of 0.83%, making it a suitable candidate for the deployment of the intrusion detection system in AVs, especially in a Mode 3 communication environment. Gossip learning (GL) provides a better approach by retaining the appropriate classifier for IDSs in AV systems [43].

## 6. Conclusions and Future Work

This paper provides a voting-based ensemble learning (VEL) and gossip learning (GL) framework for Mode 3 and Mode 4 of the V2X communication environment to detect DoS attacks on AVs using the UNSW-NB15 dataset [42].

This study contributes to the detection of DoS attacks in autonomous vehicles (AVs) by offering two distinct approaches, considering both infrastructure-based (Mode 3) and infrastructureless (Mode 4) scenarios. Unlike most existing studies, which typically focus on a single environment, this research addresses both. For Mode 3, a VEL mechanism is proposed, combining multiple classifiers to enhance detection accuracy. In contrast, for Mode 4, a graph-learning (GL) approach is employed, which is well-suited for AVs’ decentralized communication in the absence of infrastructure.

The proposed scheme’s performance is evaluated using the University of South Wales’s UNSW-NB15 dataset, from the intelligent security group. For both schemes, the proposed VEL and GL schemes, initially, the local models are trained using multiple classifiers such as ANN, LSVM, and CGSVM. In VEL, the trained model predictions are shared with the RSU, which performs voting on the received prediction outcomes and decides the ultimate output based on the majority vote. This mechanism helps to mitigate any possible bias or variance in the prediction of any individual classifier prediction, resulting in a boost in prediction performance. Then RSU shares the prediction model with the associated AVs. Since AVs also experience many situations where communication infrastructure is not available, in these cases, AVs communicate with each other. For such a situation, we provide a gossip learning framework, in which AVs communicate with each other and share their trained models, and the AVs, by themselves, decide to keep or discard a specific trained mode.

This study presents a significant advancement in the detection of various cyber-attacks, particularly denial of service (DoS) attacks, within intelligent transportation systems (ITS). Its reliability is demonstrated by a high detection rate. Moreover, this study is closely aligned with real-world scenarios by accounting for the environmental conditions experienced by autonomous vehicles (AVs). Given that DoS attacks are among the most critical types of cyber threats, the findings of this research could also be applicable to the detection of other forms of cyber attacks.

In the future, we intend to find a sophisticated ensemble method that dynamically adjusts to the environment, data distribution, and number of entities participating in the ensemble process. We also want to make ensemble methods more aligned with specific situations, where systems are required to handle big data, to enhance the scalability of ensemble learning. Ensemble learning can provide a robust interpretable mechanism, especially for critical situations, such as healthcare, disaster management, and finance. We also plan to carry out an in-depth investigation of the bias mitigation strategies within ensemble learning for various cybersecurity situations.

## Figures and Tables

**Table 1:** Statistical performance of ANN, LSVM, CGSVM, and VEL.

Parameter	CA (%)	MCR (%)	Sensitivity (%)	Specificity (%)	NPV (%)	FPR (%)	FNR (%)	Precision (%)
ANN	94.86	5.13	96.72	89.08	89.68	10.91	3.27	96.51
LSVM	97.64	2.35	98.75	91.55	93.01	8.44	1.24	91.55
CGSVM	98.82	1.17	99.73	95.03	98.86	4.96	0.26	98.80
VEL	99.16	0.83	99.35	98.12	96.66	1.87	0.006	99.64

**Table 2:** Comparison with existing approaches.

Refs. No	Article	Algorithm	Classification Accuracy
[32]	Huang K. et al. (2024)	Federated learning	98.51%
[36]	Jin Ye et al. (2018)	SVM	95.24%
[43]	Dehkordi et al. (2021)	ML + statistical methods	97.65%
[44]	Kshira S. S. et al. (2020)	SVM + GA	98.03%
[45]	Xiao-Dong Z. et al. (2019)	ACO	97.4%
[46]	Mishra A. et al. (2021)	Entropy	98.2%
[47]	Novaes et. al. (2021)	GAN	94.38%
[48]	Zhijun W. et al. (2020)	Factorization machine	95.80%
[49]	Liang W. (2022)	DFL	97%
**Our**	**GL**	**GL**	**98.82%**
**Our**	**VEL**	**VEL**	**99.16%**

## Data Availability

https://find.library.unisa.edu.au/discovery/fulldisplay/alma9916426282301831/61USOUTHAUS_INST:ROR (accessed on 15 June 2024).

## Abbreviations

The following abbreviations are used in this manuscript:

AV	Autonomous vehicle
DoS	Denial of service
RSU	Roadside units
V2X	Vehicle-to-everything
EL	Ensemble learning
GL	Gossip learning
AVS	Autonomous vehicle system
QoS	Quality of service
ITS	Intelligent transport system
ICN	Information Communication Network
V2V	Vehicle-to-vehicle
V2I	Vehicle-to-infrastructure
V2P	Vehicle-to-pedestrian
eMBB	Enhanced mobile broadband
URLLC	Ultra-reliable low-latency communication
AI	Artificial Intelligence
ML	Machine learning
DL	Deep learning
CNN	Convolutional neural network
DT	Decision tree
SVM	Support vector machine
LSVM	Linear support vector machine
CGSVM	Coarse Gaussian support vector machine
IDS	Intrusion detection system
3GPP	3rd Generation Partner Project
WAVE	Wireless ad hoc vehicle environment
PSO	Particle swarm optimization
MSE	Mean square error
ANN	Artificial neural network
ACO	Ant colony optimization
VEL	Voting-based ensemble learning
LM	Levenberg–Marquardt
BR	Bayesian Regularization
SCG	Scaled conjugate gradient
OBU	Onboard unit
IDS	Intrusion detection system
3GPP	3rd Generation Partner Project
CA	Classification accuracy
MCR	Misclassification rate
